# Factors Affecting the Breastfeeding Duration of Infants and Young Children in China: A Cross-Sectional Study

**DOI:** 10.3390/nu15061353

**Published:** 2023-03-10

**Authors:** Ziming Yang, Yingfang Ding, Shuyao Song, Yaoyun Zhang, Aolin Li, Mintao Su, Yajun Xu

**Affiliations:** 1 School of Public Health, Peking University, No. 38 Xueyuan Road, Beijing 100191, China; yangziming@pku.edu.cn (Z.Y.); 1810306139@pku.edu.cn (Y.D.); 1810306132@pku.edu.cn (S.S.); 1810306126@pku.edu.cn (Y.Z.); 1810306106@pku.edu.cn (A.L.); 1810306114@pku.edu.cn (M.S.); 2 Department of Nutrition and Food Hygiene, School of Public Health, Peking University, No. 38 Xueyuan Road, Beijing 100191, China; 3 PKUHSC-China Feihe Joint Research Institute of Nutrition and Healthy Lifespan Development, No. 38 Xueyuan Road, Beijing 100191, China; 4 Beijing Key Laboratory of Toxicological Research and Risk Assessment for Food Safety, Peking University, No. 38 Xueyuan Road, Beijing 100191, China

**Keywords:** continuous breastfeeding, breastfeeding duration, China, risk factors

## Abstract

Objective: To investigate the factors affecting the duration of continuous breastfeeding of infants within 2 years of age, and to explore intervention strategies that may promote breastfeeding duration in China. Method: A self-made electronic questionnaire was used to investigate the breastfeeding duration of infants, and the influencing factors were collected from three levels of individual, family, and social support. The Kruskal–Wallis rank sum test and the multivariable ordinal logistic regression model were used for data analysis. Subgroup analysis was carried out according to region and parity. Results: A total of 1001 valid samples from 26 provinces across the country were obtained. Among them, 9.9% breastfed for less than 6 months, 38.6% for 6 to 12 months, 31.8% for 12 to 18 months, 6.7% for 18 to 24 months, and 13.1% for more than 24 months. Barriers to sustained breastfeeding included the mother’s age at birth being over 31, education level below junior high, cesarean delivery, and the baby’s first nipple sucking at 2 to 24 h after birth. Factors that promote continued breastfeeding included freelancer or full-time mother, high breastfeeding knowledge score, supporting breastfeeding, baby with low birth weight, first bottle feeding at 4 months and later, first supplementary food at over 6 months old, high family income, the mother’s family and friends supporting breastfeeding, breastfeeding support conditions after returning to work, etc. Conclusion: The breastfeeding duration in China is generally short, and the proportion of mothers breastfeeding until the age of 2 years and above, recommended by WHO, is very low. Multiple factors at the individual, family, and social support levels influence the duration of breastfeeding. It is suggested to improve the current situation by strengthening health education, improving system security, and enhancing social support.

## 1. Introduction

Breast milk is rich in nutrients that babies need for growth and development, as well as immunoactive substances and probiotics, which promote the development of the infant’s immune system and reduce the risk of infectious diseases and allergies [1]. Breastfeeding can create an environment for emotional communication between mothers and infants, give infants a sense of security, and facilitate their psychological, behavioral, and emotional development [1]. At the same time, breastfeeding helps mothers eliminate postpartum weight retention and reduces the long-term risk of obesity, type II diabetes, heart disease, ovarian cancer, and premenopausal breast cancer [2,3]. The World Health Organization (WHO) and the United Nations International Children’s Emergency Fund (UNICEF) recommend exclusive breastfeeding for six months and continued breastfeeding until two years of age or beyond [4].

Continuous breastfeeding is defined as continuing to breastfeed on demand from six months to two years of age and beyond, which is beneficial to both moms and infants, and, while complementary feeding is introduced at 6 months of age, breast milk remains an important and safe source of nutrition [1].

Continuous breastfeeding has benefits for both infants and mothers [5]. For short-term effects, continuous breastfeeding can reduce infant mortality and prevent diseases such as diarrhea, respiratory infections, and otitis media [1,5,6]. For long-term effects, breastfeeding for a longer duration can reduce the risk of overweight and obesity and improve intelligence in children [1,5,6]. As for mothers, continuous breastfeeding can extend the birth interval and reduce the risk of breast cancer, ovarian cancer, and type II diabetes [1,5,6]. The overall breastfeeding situation in China is far from ideal, with a low breastfeeding rate and relatively short duration of breastfeeding [7,8,9,10,11,12,13]. Previous studies on factors influencing the duration of breastfeeding in China have been rare and generally limited to regional sources of samples [14,15].

Chinese Nutrition and Health Surveillance (CNHS)-2013 showed that the exclusive breastfeeding rate for children under 6 months old was 20.8%, and the continuous breastfeeding rates of 1-year-old and 2-year-old children were 11.5% and 6.9%, respectively [16], lower than the average level of developing countries [17]. There is an urgent need to identify key influencing factors to remove important barriers to breastfeeding and improve the rate and duration of breastfeeding in China.

This study investigated the influencing factors of breastfeeding duration in China from the three aspects of individual, family, and social support to provide a reference for intervention measures to promote the extension of breastfeeding duration.

## 2. Materials and Methods

### 2.1. Study Design and Participants

This cross-sectional survey was conducted from January to February 2020. In China, mothers of children aged from 6 months to 60 months were invited to participate in a web-based self-filling survey on the Wenjuanxing platform. The recruitment notices were posted and shared with the questionnaire links through WeChat Moments and groups (the most popular Chinese internet social platform). All participants gave their informed consent for inclusion before they participated in the study and received certain compensation. The study was conducted in accordance with the Declaration of Helsinki, and the protocol was approved by the Peking University Medical Ethics Committee (IRB00001052-21064).

### 2.2. Main Outcome

Breastfeeding duration was the main outcome of this study, defined as the age of infants at weaning, and was divided into 5 levels: less than 6 months, 6 to 12 months, 12 to 18 months, 18 to 24 months, and more than 24 months.

### 2.3. Questionnaire Content

The questionnaire was designed by the researchers regarding previous literature and expertise and was improved through two rounds of small sample pilot surveys to ensure that the reliability and validity were within the acceptable range.

The questionnaire included the breastfeeding duration for infants and young children, as well as the possible influencing factors of individual, family, and social support levels. The individual level included the demographic information of mothers and children, the intimate contact between mothers and infants, and mothers’ knowledge of breastfeeding (including 11 judgment questions designed concerning the previous literature [1,2,3,4,5,6,18,19,20] used to measure mothers’ breastfeeding knowledge; 1 point for each, 11 points in total; Table S1). The family level included the household monthly income per capita, marital satisfaction, and the support of family and friends for long-term breastfeeding. The social support level included the duration of maternity leave, whether maternity leave was paid, the length of working hours every day, whether there was one hour of breastfeeding time every working day, whether the workplace had a refrigerator for storing breast milk or a breastfeeding room, whether to continue breastfeeding after returning to work, the convenience of breastfeeding in public places, the breastfeeding education from medical institutions, infant formula advertisement, etc. Among them, work-related questions only need to be answered by participants with jobs, not by freelancers or full-time mothers.

The questionnaire also contained several subjective multiple-choice questions, including the participants’ breastfeeding knowledge source, the help-seeking object when encountering breastfeeding difficulties, the reasons for weaning, and the methods that they thought could promote the prolongation of breastfeeding duration.

After the questionnaires had been collected, the data integrity and rationality were checked, and those with obvious logic errors were eliminated according to the quality control demands.

### 2.4. Statistical Analysis

In the descriptive analysis, frequency and percentage were used to describe categorical variables, and mean and standard deviation for continuous variables. The outcome variable was the breastfeeding duration, which was divided into 5 levels: less than 6 months, 6 to 12 months, 12 to 18 months, 18 to 24 months, and more than 24 months. The Kruskal–Wallis rank sum test was used in the univariate analysis. The multivariable ordinal logistic regression model was used in the multivariate analysis. The breastfeeding duration was taken as the dependent variable, and the influencing factors at the three levels of individual, family, and social support as independent variables. The Akaike information criterion (AIC) was used to screen variables by stepwise regression. Samples with missing values were not filled and were eliminated in the main analysis and subgroup analyses. Since unemployed participants did not need to answer work-related questions, the logistic model was built in two parts. The first part used the full sample, and the independent variables did not contain work-related variables; the second part used the participants with jobs as the sample, and the independent variables were all variables (including work-related variables).

Subgroup analyses were carried out according to four economic zones (eastern, central, western, and northeast), divided by the National Bureau of Statistics of China, and parity (first child or second child and above). Multivariable ordinal logistic regression models were constructed respectively.

A sensitivity analysis was conducted by filling the missing values with the average level and incorporating them into the logistic regression model. The robustness of the model was described by comparing it with the main analysis results.

Microsoft Excel was used to establish the database. R 4.1.2 (R Statistics, Vienna, Austria) was used for statistical analysis, and a two-tailed *p* value < 0.05 was viewed as statistically significant.

## 3. Results

### 3.1. Participant Characteristics

A total of 1001 valid responses were collected in this survey. For mothers, the average age at birth was 26.60 ± 3.14 years old. They were from 26 provinces, autonomous regions, and municipalities in China, including 605 (60.4%) from the eastern region, 182 (18.2%) from the central region, 114 (11.4%) from the western region, and 100 (10.0%) from the northeast region. A total of 907 (90.6%) were from urban areas, and 94 cases (9.4%) were from rural areas. In addition, 33 (3.3%) were ethnic minorities. A total of 65.1% of mothers were staff in public institutions or enterprises, and the other 34.9% were freelancers or full-time mothers. A household monthly income per capita below 1000 CNY (=140 USD on 7 October 2022) accounted for 1.0%, 1000~5000 CNY (=140~702 USD) accounted for 27.8%, 5000~10,000 CNY (=702~1405 USD) accounted for 44.4%, and more than 10,000 CNY (=1405 USD) accounted for 26.9%. For children, 600 were boys (59.9%) and 401 were girls (40.1%). A total of 15.6% of children were under 24 months old, 55.7% were 24~36 months old, 23.4% were 36~48 months old, and 5.4% were 48~60 months old at the time of the survey. The average birth weight was 3.22 ± 0.54 kg. In addition, 852 (85.1%) were first born, other 149 (14.9%) were second born and above. In total, 9.9% of participants’ breastfeeding duration lasted for less than 6 months, 38.6% for 6~12 months, 31.8% for 12~18 months, 6.7% for 18~24 months, and 13.1% for more than 24 months. Detailed characteristics of the respondents are shown in Table 1.

### 3.2. Single Factor Analysis of Influencing Factors on Breastfeeding Duration

The single factor analysis results of the Kruskal–Wallis rank sum test showed that there were statistically significant differences between multiple factors and the distribution of breastfeeding duration, including mother’s age, educational background, occupation, region, breastfeeding knowledge score, self-rating of physical health during lactation, and whether the mother supports breastfeeding for over 24 months at the individual level of mothers; the child’s birth weight, the time between childbirth and the first infant and mom’s skin contact, the time between childbirth and the first nipple sucking, and the age when the milk bottle was first used at the individual level of children; monthly household income per capita, self-rating of marital satisfaction, child’s father, the elderly in the family and mother’s friends’ attitudes toward breastfeeding for over 24 months at the family level; and whether to continue breastfeeding after returning back to work, whether there was one hour of breastfeeding time every working day, whether the mother had an experience of giving up breastfeeding due to the inconvenience of breastfeeding in public, and whether they had heard or felt it was implied of the advertising that “infant formula has higher nutritional value than breast milk” at the social support level (Table 1).

### 3.3. Multivariable Ordinal Logistic Regression of Influencing Factors of Breastfeeding Duration (Main Analysis)

Risk factors for long-term breastfeeding included the mother’s age at birth being over 31 (OR = 0.45, 95%CI: 0.29~0.71) and junior high school diploma or below (OR = 0.37, 95%CI: 0.20~0.71) at the individual level of mothers; and cesarean delivery (OR = 0.73, 95%CI: 0.56~0.95) and first nipple sucking being at 2 to 24 h after birth (OR = 0.61, 95%CI: 0.46~0.79) at the individual level of children (Table 2).

Protective factors for long-term breastfeeding included the mother being a freelancer or full-time mother (OR = 2.04, 95%CI: 1.54~2.70), breastfeeding knowledge score increasing by 1 point out of 11 (OR = 1.14, 95%CI: 1.06~1.22), and supporting breastfeeding for over 24 months (OR = 2.46, 95%CI: 1.67~3.64) at the individual level of mothers; low birth weight (OR = 2.67, 95%CI: 1.69~4.23), using a milk bottle for the first time at 4–6 months old (OR = 2.40, 95%CI: 1.80~3.21) or at over 6 months old (OR = 2.28, 95%CI: 1.59~3.28), and adding supplementary food for the first time at over 6 months old (OR = 1.79, 95%CI: 1.12~2.84) at the individual level of children; monthly household income per capita being over 1405 dollars (OR = 2.03, 95%CI: 1.43~2.88) and most of the mother’s friends’ attitudes towards breastfeeding for over 24 months being uncertain rather than unsupportive (OR = 2.24, 95%CI: 1.36~3.69) at the family level; and continuous breastfeeding after returning back to work (OR = 5.32, 95%CI: 3.17~8.92), having one hour of breastfeeding time every working day (OR = 1.83, 95%CI: 1.04~3.20), and having an experience of giving up breastfeeding due to the inconvenience of breastfeeding in public (OR = 1.72, 95%CI: 1.25~2.37) at the social support level (Table 2).

### 3.4. Subgroup Analysis in Different Economic Zones and Parities

The influencing factors of breastfeeding duration were diverse in different economic zones and parities.

For the economic zones, in the east region, risk factors for long-term breastfeeding included mother’s age at birth being over 31 (OR = 0.44, 95%CI: 0.24~0.81), self-rating of physical health during lactation being lower than 6 out of 10 (OR = 0.30, 95%CI: 0.14~0.63), child’s first nipple sucking being at 2 to 24 h after birth (OR = 0.67, 95%CI: 0.47~0.96), thinking breastfeeding in public was embarrassing (OR = 0.57, 95%CI: 0.33~0.96), agreeing with the statement that “infant formula has higher nutritional value than breast milk” (OR = 0.64, 95%CI: 0.43~0.95). In contrast, protective factors included the mother being a freelancer or full-time mother (OR = 2.22, 95%CI: 1.56~3.18), living in an urban area (OR = 2.51, 95%CI: 1.34~4.73), breastfeeding knowledge score increasing by 1 point (OR = 1.11, 95%CI: 1.00~1.22), mother supporting breastfeeding for over 24 months (OR = 2.48, 95%CI: 1.32~4.67), low birth-weight child (OR = 3.88, 95%CI: 2.05~7.35), using a milk bottle for the first time at 4–6 months old (OR = 2.76, 95%CI: 1.87~4.08) or at over 6 months old (OR = 2.34, 95%CI: 1.44~3.80), adding supplementary food for the first time at 4–6 months old (OR = 2.52, 95%CI: 1.37~4.61) or at over 6 months old (OR = 3.45, 95%CI: 1.79~6.63), monthly household income per capita being over 1405 dollars (OR = 3.06, 95%CI: 1.91~4.90), self-rating of marital satisfaction being lower than 6 out of 10 (OR = 2.96, 95%CI: 1.14~7.67), child’s father supporting breastfeeding for over 24 months (OR = 2.17, 95%CI: 1.10~4.27), most of the mother’s friends’ attitudes towards breastfeeding for over 24 months being uncertain rather than unsupportive (OR = 3.99, 95%CI: 1.92~8.28), and having an experience of giving up breastfeeding due to the inconvenience of breastfeeding in public (OR = 2.22, 95%CI: 1.36~3.62) (Table 3).

In the central region, risk factors included the mother’s education level being junior high school or below (OR = 0.11, 95%CI: 0.02~0.51), living in an urban area (OR = 0.23, 95%CI: 0.06~0.81), cesarean delivery (OR = 0.49, 95%CI: 0.26~0.92), self-rating of marital satisfaction being lower than 6 (OR = 0.16, 95%CI: 0.04~0.68) or 7–8 (OR = 0.31, 95%CI: 0.13~0.72) rather than 9–10, and thinking setting up nursing rooms in public would bring convenience to nursing mothers (OR = 0.22, 95%CI: 0.06~0.77). In contrast, protective factors included self-rating of physical health during lactation being 7–8 (OR = 3.43, 95%CI: 1.55~7.61) rather than 9–10, mother supporting breastfeeding for over 24 months (OR = 3.10, 95%CI: 1.54~6.22), child using a milk bottle for the first time at 4–6 months old (OR = 2.54, 95%CI: 1.28~5.05) rather than <4 months old, and having an experience of giving up breastfeeding due to the inconvenience of breastfeeding in public (OR = 2.55, 95%CI: 1.21~5.35) (Table 3).

In the western region, risk factors included the mother supporting breastfeeding for over 24 months (OR = 0.22, 95%CI: 0.05~0.94) and the elderly in the family supporting breastfeeding for over 24 months (OR = 0.07, 95%CI: 0.02~0.34). In contrast, protective factors included breastfeeding knowledge score increasing by 1 point (OR = 1.58, 95%CI: 1.15~2.17), child’s father supporting breastfeeding for over 24 months (OR = 34.88, 95%CI: 5.38~226.07), and thinking setting up nursing rooms in public would bring convenience to nursing mothers (OR = 18.57, 95%CI: 1.94~177.80) (Table 3).

In the northeast region, protective factors included being an ethnic minority (OR = 7.31, 95%CI: 1.15~46.47), self-rating of physical health during lactation being 7–8 (OR = 5.22, 95%CI: 1.79~15.25) rather than 9–10, using a milk bottle for the first time at 4–6 months old (OR = 6.31, 95%CI: 2.05~19.41) or over 6 months old (OR = 6.22, 95%CI: 1.30~29.77), monthly household income per capita being over 1405 dollars (OR = 10.86, 95%CI: 2.32~50.86), the elderly in the family’s attitudes towards breastfeeding for over 24 months being uncertain rather than unsupportive (OR = 11.27, 95%CI: 1.20~105.42) (Table 3).

For the first child, risk factors for long-term breastfeeding included mother’s age at birth being over 31 (OR = 0.42, 95%CI: 0.24~0.75), self-rating of physical health during lactation being lower than 6 (OR = 0.60, 95%CI: 0.37~0.99), cesarean delivery (OR = 0.67, 95%CI: 0.50~0.90), and first nipple sucking being at 2 to 24 h after birth (OR = 0.56, 95%CI: 0.41~0.78) rather than <2 h. In contrast, protective factors included the mother being a freelancer or full-time mother (OR = 1.93, 95%CI: 1.43~2.61), breastfeeding knowledge score increasing by 1 point (OR = 1.12, 95%CI: 1.04~1.21), mother supporting breastfeeding for over 24 months (OR = 2.67, 95%CI: 1.73~4.13), low birth-weight child (OR = 3.02, 95%CI: 1.83~4.98), using a milk bottle for the first time at 4–6 months old (OR = 2.31, 95%CI: 1.69~3.16) or at over 6 months old (OR = 2.23, 95%CI: 1.47~3.39), adding supplementary food for the first time at over 6 months old (OR = 2.01, 95%CI: 1.20~3.35), monthly household income per capita being 702–1405 dollars (OR = 1.46, 95%CI: 1.05~2.03) or over 1405 dollars (OR = 2.43, 95%CI: 1.67~3.54), most of the mother’s friends’ attitudes towards breastfeeding for over 24 months being uncertain rather than unsupportive (OR = 2.35, 95%CI: 1.35~4.10), and having an experience of giving up breastfeeding due to the inconvenience of breastfeeding in public (OR = 1.78, 95%CI: 1.24~2.54) (Table 4).

For the second child or above, risk factors included first nipple sucking being at 2 to 24 h after birth (OR = 0.35, 95%CI: 0.16~0.78) rather than <2 h and monthly household income per capita being 702–1405 dollars (OR = 0.39, 95%CI: 0.16~0.94) rather than <702 dollars. In contrast, protective factors included living in an urban area (OR = 5.45, 95%CI: 1.84~16.16), mother supporting breastfeeding for over 24 months (OR = 4.86, 95%CI: 1.78~13.29), using a milk bottle for the first time at over 6 months old (OR = 2.90, 95%CI: 1.14~7.37), the elderly in the family’s attitudes towards breastfeeding for over 24 months being uncertain rather than unsupportive (OR = 12.97, 95%CI: 2.20~76.58), and thinking breastfeeding in public was embarrassing (OR = 5.26, 95%CI: 1.99~13.95) (Table 4).

### 3.5. Sensitivity Analysis

The sensitivity analysis was basically consistent with the main analysis results, indicating the regression models were robust. See Table S2 for detailed sensitivity analysis results.

### 3.6. Answers to the Subjective Questions

The top five reasons for weaning among the respondents included fearing their children were too dependent on them and would lack independence in the future (40.3%), considering the nutritional value of breast milk was not high (33.1%), the reduction or absence of their breast milk (24.5%), continuous breastfeeding would continue to replenish nutrition, which was not conducive to their weight recovery (15.3%), and considering breastfeeding every day was too hard (14.8%).

The top three sources of the respondents’ breastfeeding knowledge were medical institutions (80.2%), networks (58.1%), and posters in public places other than hospitals (50.3%).

The top three subjects for help when the respondents encountered breastfeeding difficulties after giving birth were doctors and nurses (68.0%), breast therapists (54.8%), family and friends (47.6%), care workers (32.0%), and networks (31.3%).

A total of 112 respondents with jobs did not continue breastfeeding after returning to work from maternity leave. The top three reasons included long hours and high intensity of work (51.2%), reduction of breast milk (50.1%), and no breastfeeding room or refrigerator to store breast milk at the workplace (48.2%).

The top five ways that could promote the prolongation of breastfeeding duration believed by respondents were to set up breastfeeding rooms in public places (65.6%), to extend the duration of paid maternity leave (61.3%), to set up refrigerators to store breast milk at workplaces (60.5%), to increase publicity on the advantages of breastfeeding from communities and hospitals (59.9%), and to increase publicity of them from online media (41.0%).

## 4. Discussion

This survey showed that the breastfeeding duration of children in China was mainly from 6 to 12 months (38.6%), followed by 12 to 18 months (31.8%), which was similar to the results of Wang et al. [21] and slightly higher than the results of Li et al. [22] and Liu et al. [23], but still far shorter from the WHO recommendation of continuous breastfeeding to 2 years old or above. Important aspects that influence the duration of breastfeeding in China were as below.

On the aspect of mothers, the breastfeeding duration of mothers over 31 years of age was significantly shorter than that of mothers under 30 years of age. This may be related to the fact that women over 30 years of age may have higher positions at work and greater social responsibility and work pressure. It is worth mentioning that the age range of mothers found in this study in favor of continuous breastfeeding was also close to the optimal reproductive age of women found in studies at home and abroad [24,25], suggesting that an appropriate childbearing age can not only reduce the risk of pregnancy complications and adverse pregnancy outcomes but also have a positive impact on the breastfeeding duration. Healthy mothers breastfeed longer, which was consistent with a previous study [26]. Therefore, strengthening health education for women of childbearing age, sex education for adolescents, and reducing the occurrence of infectious and chronic diseases in pregnant women can be beneficial for promoting breastfeeding. Mothers with correct knowledge of breastfeeding had significantly longer breastfeeding duration, consistent with previous studies [19,27]. Mothers who supported long-term breastfeeding had breastfed for a significantly longer time, and a previous study had also shown that mothers’ perceptions, attitudes, and confidence were important for the breastfeeding duration [22]. Subgroup analysis showed that knowledge about breastfeeding had the greatest impact on the duration of breastfeeding in the western region of China, followed by the central region and the eastern region. Therefore, it is of crucial importance to strengthen the publicity of breastfeeding knowledge and establish the correct concept and attitude of pregnant women towards breastfeeding, especially in less developed areas. The breastfeeding duration of mothers with high education level and high family income was relatively longer, consistent with the results of Su et al. [28]. The reason may be that mothers with high education and good economic conditions can better understand the importance of breastfeeding. In the eastern region, children from cities, instead of the countryside, had been breastfeeding for longer, while the opposite was true in the central region. This might be because, with the rapid development of the central region in recent years, people from cities had relatively good economic conditions, but they still had an insufficient understanding of the importance of breastfeeding and added formula milk and complementary foods prematurely. Therefore, improving the education level of women in less developed areas may help to promote the extension of breastfeeding duration. Meanwhile, the breastfeeding duration of second-born infants from cities was longer than those from rural areas, which may also be related to the level of the economy, education, and mothers’ breastfeeding knowledge, suggesting that it is needed to pay attention to breastfeeding guidance of second-born infants in rural areas.

On the aspect of children, this study showed that the breastfeeding duration was longer for low-weight children, but this was inconsistent with the results of Su et al. [29], which may be related to the different backgrounds of the subjects. The breastfeeding duration in children delivered by cesarean section was shorter than in those delivered vaginally, consistent with previous studies [26]. The breastfeeding duration was significantly longer in infants who had the first nipple sucking within 2 h, using a bottle after 4 months of age, and adding complementary food after 6 months of age, consistent with previous studies [21,22,28,30]. Therefore, in health education, attention should be paid to the early initiation of breastfeeding, using of bottles not too early, and age-appropriate addition of supplementary food knowledge publicity, as well as the promotion of vaginal delivery and reducing the number of non-indicative cesarean section deliveries, which would be beneficial to promote continuous breastfeeding.

On the aspect of family, high family income and the support for continuous breastfeeding from the father of the child and friends of the mothers were conducive to the promotion of long-term breastfeeding, consistent with the results of domestic and foreign studies [31,32]. In the western region, however, mothers who receive support from the family elders breastfeed for less time. This might be related to the low education level of the elderly in the western region, which led to the mother’s distrust of their childcare concepts. At the same time, the present survey showed that when the mother encountered lactation difficulties, in addition to the professional medical personnel and milk practitioners, most of the consultation was offered by family members and friends. Therefore, it is suggested that the object of breastfeeding health education can be extended to the pregnant women’s spouses, parents, and friends to form a positive breastfeeding support environment.

On the aspect of social support, for work-related factors, the breastfeeding duration was significantly shorter for mothers who work in enterprises and public institutions compared with freelancers or stay-at-home mothers, consistent with previous research results [22,33], possibly because going out to work would lead to separation of mothers and infants [34]. In this study, mothers who had one hour of breastfeeding time on weekdays had significantly longer breastfeeding duration. Mothers who continued to breastfeed their children after returning to work from maternity leave had significantly longer breastfeeding duration, consistent with the research results of Zhang et al. [35]. Our survey also revealed that long working hours, high intensity, and the lack of a lactation room or refrigerator to store breast milk were the main reasons for not continuing to breastfeed after resuming work according to the answers of participants. Meanwhile, previous studies had also shown that excessive work intensity and pressure were not conducive to breastfeeding [36]. Therefore, it is suggested that work units should protect the basic rights and interests of pregnant female workers, give more care and preferential treatment, give certain flexible working hours and appropriate work intensity to those who have just resumed work, and suggest the provision of breastfeeding rooms and special refrigerators for storing breast milk.

As for the factors of medical institutions, there was no significant difference between the breastfeeding education provided by professionals during pregnancy and childbirth and the breastfeeding duration, indicating that the effect of breastfeeding education was not ideal. It is suggested that medical institutions pay more attention to the standardization and professional training of health education personnel to correctly and effectively transmit health education information.

As for other social factors, according to our survey, 73.9% of respondents had once given up breastfeeding their children due to the inconvenience of breastfeeding in public places, 82.3% thought it was embarrassing to breastfeed in public places, and 96.3% thought setting up breastfeeding rooms in public places could bring convenience to nursing mothers. Therefore, it is important to set up lactation rooms in public places and increase the public’s tolerance to breastfeeding mothers in public places. As for the advertising of infant formula, 73.5% of the respondents had heard or felt it was implied that the nutritional value of infant formula was higher than that of breast milk, and 38.2% agreed with the statement that the nutritional value of infant formula was higher than that of breast milk. A previous study [28] also showed that the vigorous promotion of milk substitutes might reduce the breastfeeding rate. It is suggested to strengthen market supervision and prohibit infant formula enterprises from advertising with improper wording. At the same time, our study showed that a high proportion of respondents learned breastfeeding knowledge and sought help from the internet. Therefore, under the premise of strictly ensuring scientific knowledge, the way of breastfeeding health education can be presented by new media to make it more vivid, and the publicity of breastfeeding on the internet platform and mothers’ community can be strengthened.

This study has certain limitations. As a retrospective survey, there may be recall bias in the information provided by the participants. However, to minimize this bias, we recruited mothers with children not older than 60 months. In addition, because the questionnaires were collected through the internet, the participants were all internet users, which may lead to certain selection bias. However, according to the 47th China Statistical Report on Internet Development and China Statistical Yearbook, in 2020, the proportion of internet users among the population aged 20–49 in China reached 94.6% (participants in this study are aged 20–46), and WeChat monthly active users reached 1.203 billion in 2020, covering 85.3% of the Chinese population.

## 5. Conclusions

By the beginning of 2020, the breastfeeding duration in China was still not ideal. The economic level, breastfeeding knowledge, family support, work factors, and social support were all key influencing factors, and there were differences in geographical regions and parities. It is suggested to improve the content of breastfeeding health education, expand the scope of the audience, promote the new media communication mode, promote the training and specialization of health education personnel, protect the rights and interests of female workers, standardize infant formula advertising and carry out effective supervision, and widely improve social support. In addition, more targeted interventions can be implemented according to the heterogeneity of influencing factors of breastfeeding in different regions and parities to jointly promote the extension of the breastfeeding duration in China.

## Figures and Tables

**Table 1:** Respondent characteristics (*n* = 1001) and single factor analysis of influencing factors on breastfeeding duration.

Factor	N	Breastfeeding Duration (Months, %)					χ^2^	*p* Value
		0–6	6–12	12–18	18–24	Over 24		
**Individual Level of Mothers**								
Age ^a^							28.53	<0.001
≤25	360	8.6	37.5	33.6	6.9	13.3		
26–30	493	7.9	37.1	33.3	7.5	14.2		
≥31	102	22.5	47.1	21.6	4.9	3.9		
Nation							0.03	0.856
Ethnic Han	968	10.0	38.3	31.8	6.6	13.2		
Ethnic minorities	33	6.1	45.5	30.3	9.1	9.1		
Educational background							15.93	0.001
Bachelor or postgraduate	434	8.8	35.9	35.3	7.1	12.9		
Junior college	403	8.4	41.2	31.5	6.9	11.9		
Senior high school	112	12.5	34.8	27.7	3.6	21.4		
Junior high school or below	52	25.0	48.1	13.5	7.7	5.8		
Occupation							8.73	0.003
Staff of public institutions and enterprises	652	11.0	37.9	36.2	6.7	8.1		
Freelancer or full-time mother	349	7.7	39.8	23.5	6.6	22.3		
Region							4.55	0.033
Rural	94	13.8	47.9	20.2	3.2	14.9		
Urban	907	9.5	37.6	33.0	7.1	12.9		
Economic zone							7.78	0.051
Eastern region	605	11.4	37.2	29.1	6.4	15.9		
Central region	182	9.3	38.5	36.3	6.6	9.3		
Western region	114	7.0	53.5	25.4	6.1	7.9		
Northeast region	100	5.0	30.0	47.0	9.0	9.0		
Breastfeeding knowledge score (composed of 11 judgment questions) ^b^							75.84	<0.001
≤5	119	16.0	53.8	23.5	1.7	5.0		
6	108	11.1	50.9	25.9	2.8	9.3		
7	220	12.7	42.3	35.0	5.9	4.1		
8	198	6.6	35.4	25.8	6.1	26.3		
9	129	11.6	33.3	31.8	9.3	14.0		
10	118	8.5	25.4	41.5	11.9	12.7		
11	109	1.8	28.4	40.4	10.1	19.3		
Self-rating of mental health during lactation (out of 10)							3.16	0.206
9–10	601	8.7	39.1	32.9	7.8	11.5		
7–8	312	9.9	37.8	31.1	4.8	16.3		
≤6	88	18.2	37.5	26.1	5.7	12.5		
Self-rating of physical health during lactation (out of 10)							9.29	0.010
9–10	620	8.2	39.2	33.2	7.1	12.3		
7–8	291	11.0	35.1	32.0	5.8	16.2		
≤6	90	17.8	45.6	21.1	6.7	8.9		
Whether the mother supports breastfeeding for over 24 months							77.78	<0.001
Not support	352	12.5	53.4	28.4	2.0	3.7		
Support	649	8.5	30.5	33.6	9.2	18.2		
**Individual Level of Children**								
Gender							0.28	0.596
Male	600	9.8	38.2	33.8	7.5	10.7		
Female	401	10.0	39.2	28.7	5.5	16.7		
Birth weight ^c^							10.76	0.005
Normal birth weight (2500–4000 g)	824	9.2	39.1	33.3	6.8	11.7		
Low birth weight (<2500 g)	85	8.2	31.8	25.9	4.7	29.4		
Macrosomia (>4000 g)	73	17.8	39.7	26.0	9.6	6.8		
Parity							0.97	0.324
First child	852	9.7	38.3	31.8	6.2	14.0		
Second child or above	149	10.7	40.3	31.5	9.4	8.1		
Delivery mode							0.84	0.360
Vaginal delivery	379	11.1	36.1	29.3	8.7	14.8		
Cesarean delivery	622	9.2	40.0	33.3	5.5	12.1		
Gestational age							0.07	0.791
Full-term (≥37 weeks)	747	9.5	39.4	31.3	7.6	12.2		
Preterm (<37 weeks)	254	11.0	36.2	33.1	3.9	15.7		
Time between childbirth and the first infant and mom’s skin contact							8.65	0.013
<2 h	542	9.4	36.9	29.3	8.1	16.2		
2–24 h	430	9.5	40.9	34.9	5.1	9.5		
>24 h	29	24.1	34.5	31.0	3.4	6.9		
Time between childbirth and the first nipple sucking							24.59	<0.001
<2 h	413	9.4	31.2	31.7	9.2	18.4		
2–24 h	508	10.6	44.9	30.3	5.1	9.1		
>24 h	80	7.5	36.2	41.2	3.8	11.2		
Age when the milk bottle was first used							54.19	<0.001
<4 months	439	15.5	42.8	30.3	6.4	5.0		
4–6 months	398	6.8	33.7	33.7	5.5	20.4		
>6 months	164	2.4	39.0	31.1	10.4	17.1		
Age when supplementary food was added							5.51	0.064
<4 months	121	13.2	40.5	37.2	2.5	6.6		
4–6 months	526	10.1	37.1	33.5	6.5	12.9		
>6 months	354	8.5	40.1	27.4	8.5	15.5		
**Family Level**								
Monthly household income per capita ^d^							25.05	<0.001
<5000 CNY (<702 USD)	288	10.8	46.9	29.5	7.6	5.2		
5000–10,000 CNY (702–1405 USD)	444	11.5	34.7	36.0	6.1	11.7		
>10,000 CNY (>1405 USD)	269	6.3	36.1	27.1	6.7	23.8		
Self-rating of marital satisfaction (out of 10)							9.63	0.008
9–10	717	7.9	37.8	33.3	7.7	13.2		
7–8	236	14.4	42.4	26.7	3.8	12.7		
≤6	48	16.7	31.2	33.3	6.2	12.5		
Whether the child’s father supports breastfeeding for over 24 months							78.46	<0.001
Not support	363	13.2	52.9	25.9	1.9	6.1		
Uncertain	52	13.5	30.8	42.3	5.8	7.7		
Support	586	7.5	30.4	34.5	9.7	17.9		
Whether the elderly in the family support breastfeeding for over 24 months							27.47	<0.001
Not support	340	8.8	52.4	26.5	2.9	9.4		
Uncertain	53	13.2	34.0	41.5	9.4	1.9		
Support	608	10.2	31.2	33.9	8.6	16.1		
Whether most of the mother’s friends support breastfeeding for over 24 months							67.92	<0.001
Not support	380	11.6	53.2	26.3	3.2	5.8		
Uncertain	90	13.3	28.9	40.0	7.8	10.0		
Support	531	8.1	29.8	34.3	9.0	18.8		
**Social Support Level**								
Whether the mother received professional breastfeeding education during pregnancy							0.32	0.574
No	291	10.3	38.5	33.3	6.5	11.3		
Yes	710	9.7	38.6	31.1	6.8	13.8		
Duration of maternity leave (months)							6.76	0.239
≥6	165	17.0	31.5	33.3	7.9	10.3		
5	65	6.2	41.5	43.1	6.2	3.1		
4	64	18.8	34.4	25.0	14.1	7.8		
3	212	6.6	40.1	40.6	7.5	5.2		
2	107	7.5	37.4	40.2	0.9	14.0		
≤1	39	15.4	53.8	20.5	2.6	7.7		
Whether maternity leave was paid							1.23	0.540
Full pay	365	11.8	34.2	38.6	7.4	7.9		
Partially paid	258	9.7	43.4	33.3	5.0	8.5		
No pay	29	13.8	34.5	31.0	13.8	6.9		
Working hours per day							0.29	0.590
<8 h	411	11.4	38.0	36.3	7.1	7.3		
>8 h	241	10.4	37.8	36.1	6.2	9.5		
Whether to continue breastfeeding after returning to work							44.92	<0.001
No	112	32.1	40.2	20.5	2.7	4.5		
Yes	540	6.7	37.4	39.4	7.6	8.9		
How to breastfeed after returning to work							7.71	0.052
Breastfeeding in the workplace	73	11.0	37.0	34.2	12.3	5.5		
Go home to breastfeed after getting off work	82	4.9	39.0	43.9	7.3	4.9		
Use a breast pump or hand to express milk and take home	170	10.6	40.0	34.1	4.7	10.6		
Round-trip home in person to breastfeed	213	2.8	35.2	43.2	8.5	10.3		
Whether there was one hour of breastfeeding time every working day							26.61	<0.001
No	90	25.6	45.6	22.2	2.2	4.4		
Yes	562	8.7	36.7	38.4	7.5	8.7		
Whether a nursing room was set up in the workplace or nearby area							0.00	0.980
No	233	12.4	36.1	36.1	6.0	9.4		
Yes	419	10.3	38.9	36.3	7.2	7.4		
Whether the workplace had a refrigerator to store the breast milk							2.56	0.109
No	229	13.1	40.6	31.9	5.7	8.7		
Yes	423	9.9	36.4	38.5	7.3	7.8		
Whether the mother had an experience of giving up breastfeeding due to the inconvenience of breastfeeding in public							6.19	0.013
No	261	11.5	45.2	24.5	7.3	11.5		
Yes	740	9.3	36.2	34.3	6.5	13.6		
Whether to think breastfeeding in public was embarrassing							0.12	0.733
No	177	10.2	38.4	28.8	7.3	15.3		
Yes	824	9.8	38.6	32.4	6.6	12.6		
Whether to think setting up nursing rooms in public would bring convenience to nursing mothers							0.34	0.560
No	37	10.8	43.2	27.0	8.1	10.8		
Yes	964	9.9	38.4	32.0	6.6	13.2		
Whether they had heard or felt it was implied of the advertising that “infant formula has higher nutritional value than breast milk”							5.12	0.024
No	265	9.8	44.5	30.2	6.4	9.1		
Yes	736	9.9	36.4	32.3	6.8	14.5		
Whether they agreed with the statement that “infant formula has higher nutritional value than breast milk”							0.39	0.535
No	619	8.6	39.4	32.0	8.1	12.0		
Yes	382	12.0	37.2	31.4	4.5	14.9		

CNY: China Yuan; USD: United States dollar. ^a^ 46 missing values not shown. ^b^ See Table S1 for detailed 11 judgment questions about breastfeeding knowledge summarized by researchers based on the previous literature. ^c^ 19 missing values not shown. ^d^ 1 CNY = 0.1405 USD on 7 October 2022.

**Table 2:** Multivariable ordinal logistic regression of influencing factors of breastfeeding duration (main analysis).

Factor	Full Sample, without Work-Related Variables (n = 942) ^a^		Samples with Jobs, with All Variables (n = 622) ^b^	
	*p* Value	OR (95%CI)	*p* Value	OR (95%CI)
**Individual Level of Mothers**				
Mother’s age (vs. ≤25)				
26–30	0.562	1.08 (0.83~1.40)	0.006	0.62 (0.44~0.87)
≥31	<0.001	0.45 (0.29~0.71)	<0.001	0.38 (0.22~0.66)
Mother is an ethnic minority (vs. ethnic Han)	0.101	1.83 (0.89~3.76)	-	-
Mother’s educational background (vs. bachelor or postgraduate)				
Junior college	0.195	0.83 (0.63~1.10)	0.855	1.03 (0.74~1.43)
Senior high school	0.495	0.86 (0.55~1.33)	0.376	0.74 (0.37~1.45)
Junior high school or below	0.003	0.37 (0.20~0.71)	<0.001	0.20 (0.08~0.51)
Mother is a freelancer or full-time mother (vs. staff of public institutions and enterprises)	<0.001	2.04 (1.54~2.70)	/	/
Mother’s breastfeeding knowledge score (out of 11)	<0.001	1.14 (1.06~1.22)	0.090	1.08 (0.99~1.18)
Self-rating of physical health during lactation (out of 10, vs. 9–10)				
7–8	-	-	0.381	0.85 (0.60~1.22)
≤6	-	-	0.025	0.52 (0.30~0.92)
Mother supports breastfeeding for over 24 months (vs. not support)	<0.001	2.46 (1.67~3.64)	<0.001	2.37 (1.47~3.83)
**Individual Level of Children**				
Child’s birth weight (vs. normal birth weight)				
Low birth weight (<2500 g)	<0.001	2.67 (1.69~4.23)	0.026	2.11 (1.09~4.08)
Macrosomia (>4000 g)	0.813	0.94 (0.58~1.54)	0.334	0.74 (0.41~1.35)
Cesarean delivery (vs. vaginal delivery)	0.017	0.73 (0.56~0.95)	0.001	0.59 (0.42~0.81)
Time between childbirth and the first nipple sucking (vs. <2 h)				
2–24 h	<0.001	0.61 (0.46~0.79)	0.025	0.68 (0.49~0.95)
>24 h	0.274	1.31 (0.81~2.11)	0.639	1.16 (0.63~2.13)
Child’s age when the milk bottle was first used (vs. <4 months)				
4–6 months	<0.001	2.40 (1.80~3.21)	<0.001	2.22 (1.56~3.16)
>6 months	<0.001	2.28 (1.59~3.28)	<0.001	2.76 (1.74~4.39)
Child’s age when supplementary food was added (vs. <4 months)				
4–6 months	0.065	1.50 (0.97~2.30)	-	-
>6 months	0.014	1.79 (1.12~2.84)	-	-
**Family Level**				
Monthly household income per capita (vs. <702 USD)				
702–1405 USD	0.176	1.23 (0.91~1.68)	-	-
>1405 USD	<0.001	2.03 (1.43~2.88)	-	-
Whether most of the mother’s friends support breastfeeding for over 24 months (vs. not support)				
Uncertain	0.002	2.24 (1.36~3.69)	0.006	2.47 (1.30~4.70)
Support	0.063	1.43 (0.98~2.07)	0.215	1.33 (0.85~2.11)
**Social Support Level**				
Once received professional breastfeeding education during pregnancy	-	-	0.004	0.55 (0.37~0.82)
Continuous breastfeeding after returning back to work	/	/	<0.001	5.32 (3.17~8.92)
Had one hour of breastfeeding time every working day	/	/	0.035	1.83 (1.04~3.20)
A nursing room was set up in the workplace or nearby area	/	/	0.066	0.70 (0.48~1.02)
Had an experience of giving up breastfeeding due to the inconvenience of breastfeeding in public	<0.001	1.72 (1.25~2.37)	<0.001	2.48 (1.63~3.77)
Agreed with the statement that “infant formula has higher nutritional value than breast milk”	0.114	0.78 (0.58~1.06)	0.003	0.56 (0.39~0.83)

OR: adjusted odds ratio; CI: confidence interval; USD: United States dollar. ^a^ McFadden’s R^2^ = 0.11. ^b^ McFadden’s R² = 0.14. - for variables without statistical significance that were removed by stepwise regression; / for not applicable.

**Table 3:** Multivariable ordinal logistic regression of influencing factors of breastfeeding duration in different economic zones (subgroup analysis).

Factor	Eastern Region (n = 566) ^a^		Central Region (n = 178) ^b^		Western Region (n = 102) ^c^		Northeast Region (n = 96) ^d^	
	*p* Value	OR (95%CI)	*p* Value	OR (95%CI)	*p* Value	OR (95%CI)	*p* Value	OR (95%CI)
**Individual Level of Mothers**								
Mother’s age (vs. ≤25)								
26–30	0.306	1.20 (0.84~1.71)	-	-	-	-	-	-
≥31	0.008	0.44 (0.24~0.81)	-	-	-	-	-	-
Mother is an ethnic minority (vs. ethnic Han)	-	-	-	-	-	-	0.035	7.31 (1.15~46.47)
Mother’s educational background (vs. bachelor or postgraduate)								
Junior college	-	-	0.069	0.54 (0.28~1.05)	-	-	-	-
Senior high school	-	-	0.933	0.96 (0.33~2.76)	-	-	-	-
Junior high school or below	-	-	0.005	0.11 (0.02~0.51)	-	-	-	-
Mother is a freelancer or full-time mother (vs. staff of public institutions and enterprises)	<0.001	2.22 (1.56~3.18)	0.124	1.70 (0.86~3.33)	-	-	-	-
Urban (vs. rural)	0.004	2.51 (1.34~4.73)	0.022	0.23 (0.06~0.81)	-	-	-	-
Mother’s breastfeeding knowledge score (out of 11)	0.040	1.11 (1.00~1.22)	0.053	1.20 (1.00~1.45)	0.005	1.58 (1.15~2.17)	-	-
Self-rating of physical health during lactation (out of 10, vs. 9–10)								
7–8	0.286	0.81 (0.56~1.19)	0.002	3.43 (1.55~7.61)	-	-	0.003	5.22 (1.79~15.25)
≤6	0.002	0.30 (0.14~0.63)	0.403	1.66 (0.51~5.45)	-	-	0.202	2.66 (0.59~11.97)
Mother supports breastfeeding for over 24 months (vs. not support)	0.005	2.48 (1.32~4.67)	0.001	3.10 (1.54~6.22)	0.041	0.22 (0.05~0.94)	-	-
**Individual Level of Children**								
Child’s birth weight (vs. normal birth weight)								
Low birth weight (<2500 g)	<0.001	3.88 (2.05~7.35)	-	-	-	-	-	-
Macrosomia (>4000 g)	0.938	0.97 (0.51~1.87)	-	-	-	-	-	-
Cesarean delivery (vs. vaginal delivery)	-	-	0.027	0.49 (0.26~0.92)	-	-	-	-
Time between childbirth and the first infant and mom’s skin contact (vs. <2 h)								
2–24 h	-	-	-	-	-	-	0.031	2.73 (1.09~6.81)
>24 h	-	-	-	-	-	-	<0.001	0.00 (0.00~0.00)
Time between childbirth and the first nipple sucking (vs. <2 h)								
2–24 h	0.027	0.67 (0.47~0.96)	0.110	0.58 (0.30~1.13)	-	-	-	-
>24 h	0.774	0.90 (0.44~1.83)	0.154	2.09 (0.76~5.73)	-	-	-	-
Child’s age when the milk bottle was first used (vs. <4 months)								
4–6 months	<0.001	2.76 (1.87~4.08)	0.008	2.54 (1.28~5.05)	-	-	0.001	6.31 (2.05~19.41)
>6 months	<0.001	2.34 (1.44~3.80)	0.127	2.03 (0.82~5.03)	-	-	0.022	6.22 (1.30~29.77)
Child’s age when supplementary food was added (vs. <4 months)								
4–6 months	0.003	2.52 (1.37~4.61)	-	-	0.179	0.34 (0.07~1.64)	-	-
>6 months	<0.001	3.45 (1.79~6.63)	-	-	0.622	1.50 (0.30~7.50)	-	-
**Family Level**								
Monthly household income per capita (vs. <702 USD)								
702–1405 USD	0.056	1.53 (0.99~2.36)	0.058	1.99 (0.98~4.07)	-	-	0.357	1.64 (0.57~4.69)
>1405 USD	<0.001	3.06 (1.91~4.90)	0.936	0.96 (0.39~2.36)	-	-	0.002	10.86 (2.32~50.86)
Self-rating of marital satisfaction (out of 10, vs. 9–10)								
7–8	0.572	0.88 (0.56~1.37)	0.007	0.31 (0.13~0.72)	-	-	-	-
≤6	0.026	2.96 (1.14~7.67)	0.013	0.16 (0.04~0.68)	-	-	-	-
Whether the child’s father supports breastfeeding for over 24 months (vs. not support)								
Uncertain	0.059	2.71 (0.96~7.60)	-	-	0.726	0.73 (0.13~4.19)	-	-
Support	0.025	2.17 (1.10~4.27)	-	-	<0.001	34.88 (5.38~226.07)	-	-
Whether the elderly in the family support breastfeeding for over 24 months (vs. not support)								
Uncertain	-	-	-	-	0.093	0.21 (0.03~1.30)	0.034	11.27 (1.20~105.42)
Support	-	-	-	-	<0.001	0.07 (0.02~0.34)	0.053	4.50 (0.98~20.56)
Whether most of the mother’s friends support breastfeeding for over 24 months (vs. not support)								
Uncertain	<0.001	3.99 (1.92~8.28)	-	-	-	-	0.065	0.18 (0.03~1.12)
Support	0.548	1.18 (0.69~2.04)	-	-	-	-	0.154	0.34 (0.08~1.50)
**Social Support Level**								
Once received professional breastfeeding education during pregnancy	-	-	-	-	0.133	2.02 (0.81~5.05)	-	-
Had an experience of giving up breastfeeding due to the inconvenience of breastfeeding in public	0.001	2.22 (1.36~3.62)	0.013	2.55 (1.21~5.35)	-	-	-	-
Think breastfeeding in public was embarrassing	0.036	0.57 (0.33~0.96)	-	-	0.084	2.41 (0.89~6.54)	-	-
Think setting up nursing rooms in public would bring convenience to nursing mothers	-	-	0.018	0.22 (0.06~0.77)	0.011	18.57 (1.94~177.80)		
Agreed with the statement that “infant formula has higher nutritional value than breast milk”	0.029	0.64 (0.43~0.95)	-	-	-	-	-	-

OR: adjusted odds ratio; CI: confidence interval; USD: United States dollar. ^a^ McFadden’s R^2^ = 0.18. ^b^ McFadden’s R^2^ = 0.17. ^c^ McFadden’s R^2^ = 0.15. ^d^ McFadden’s R^2^ = 0.22. - for variables without statistical significance that were removed by stepwise regression.

**Table 4:** Multivariable ordinal logistic regression of influencing factors of breastfeeding duration in different parities (subgroup analysis).

Factor	First Child (n = 805) ^a^		Second Child or Above (n = 137) ^b^	
	*p* Value	OR (95%CI)	*p* Value	OR (95%CI)
**Individual Level of Mothers**				
Mother’s age (vs. ≤25)				
26–30	0.587	1.08 (0.82~1.43)	-	-
≥31	0.003	0.42 (0.24~0.75)	-	-
Mother is an ethnic minority (vs. ethnic Han)	0.103	1.96 (0.87~4.38)	-	-
Mother’s educational background (vs. bachelor or postgraduate)				
Junior college	-	-	0.501	0.73 (0.30~1.81)
Senior high school	-	-	0.140	2.34 (0.76~7.26)
Junior high school or below	-	-	0.201	0.40(0.10~1.63)
Mother is a freelancer or full-time mother (vs. staff of public institutions and enterprises)	<0.001	1.93 (1.43~2.61)	0.118	1.98 (0.84~4.67)
Urban (vs. rural)	-	-	0.002	5.45 (1.84~16.16)
Economic zone (vs. eastern region)				
Central region	-	-	0.143	2.22 (0.76~6.44)
Western region	-	-	0.152	0.49 (0.18~1.30)
Northeast region	-	-	0.124	3.73 (0.70~19.99)
Mother’s breastfeeding knowledge score (out of 11)	0.004	1.12 (1.04~1.21)	0.064	1.26 (0.99~1.60)
Self-rating of physical health during lactation (out of 10, vs. 9–10)				
7–8	0.717	1.06 (0.78~1.42)	-	-
≤6	0.047	0.60 (0.37~0.99)	-	-
Mother supports breastfeeding for over 24 months (vs. not support)	<0.001	2.67 (1.73~4.13)	0.002	4.86 (1.78~13.29)
**Individual Level of Children**				
Child’s birth weight (vs. normal birth weight)				
Low birth weight (<2500 g)	<0.001	3.02 (1.83~4.98)	-	-
Macrosomia (>4000 g)	0.807	0.94 (0.56~1.58)	-	-
Cesarean delivery (vs. vaginal delivery)	0.007	0.67 (0.50~0.90)	-	-
Premature delivery (vs. full-term delivery)	-	-	0.064	0.38 (0.14~1.06)
Time between childbirth and the first infant and mom’s skin contact (vs. <2 h)				
2–24 h	0.061	1.35 (0.99~1.85)	-	-
>24 h	0.132	0.50 (0.20~1.24)	-	-
Time between childbirth and the first nipple sucking (vs. <2 h)				
2–24 h	<0.001	0.56 (0.41~0.78)	0.010	0.35 (0.16~0.78)
>24 h	0.593	1.18 (0.65~2.13)	0.569	1.39 (0.45~4.31)
Child’s age when the milk bottle was first used (vs. <4 months)				
4–6 months	<0.001	2.31 (1.69~3.16)	0.124	2.02 (0.83~4.92)
>6 months	<0.001	2.23 (1.47~3.39)	0.025	2.90 (1.14~7.37)
Child’s age when supplementary food was added (vs. <4 months)				
4–6 months	0.058	1.58 (0.98~2.55)	-	-
>6 months	0.008	2.01 (1.20~3.35)	-	-
**Family Level**				
Monthly household income per capita (vs. <702 USD)				
702–1405 USD	0.024	1.46 (1.05~2.03)	0.035	0.39 (0.16~0.94)
>1405 USD	<0.001	2.43 (1.67~3.54)	0.399	1.59 (0.54~4.65)
Whether the elderly in the family support breastfeeding for over 24 months (vs. not support)				
Uncertain	-	-	0.005	12.97 (2.20~76.58)
Support	-	-	0.076	2.09 (0.93~4.71)
Whether most of the mother’s friends support breastfeeding for over 24 months (vs. not support)				
Uncertain	0.003	2.35 (1.35~4.10)	-	-
Support	0.081	1.44 (0.96~2.18)	-	-
**Social Support Level**				
Once received professional breastfeeding education during pregnancy	-	-	0.051	0.45 (0.20~1.00)
Had an experience of giving up breastfeeding due to the inconvenience of breastfeeding in public	0.002	1.78 (1.24~2.54)	-	-
Think breastfeeding in public was embarrassing	-	-	<0.001	5.26 (1.99~13.95)
Agreed with the statement that “infant formula has higher nutritional value than breast milk”	0.081	0.75 (0.54~1.04)	-	-

OR: adjusted odds ratio; CI: confidence interval; USD: United States dollar. ^a^ McFadden’s R^2^ = 0.12. ^b^ McFadden’s R^2^ = 0.22. - for variables without statistical significance that were removed by stepwise regression.

## Data Availability

The data presented in this study are available on reasonable request from the corresponding author.

## Supplementary Materials

The following supporting information can be downloaded at: https://www.mdpi.com/article/10.3390/nu15061353/s1, Table S1: Eleven judgment questions used to measure mothers’ breastfeeding knowledge (1 point for each, 11 points in total) and their single factor analysis results, Table S2: Sensitivity analysis results for the multivariable ordinal logistic regression of influencing factors of breastfeeding duration.

## References

[1] UNICEF (2016). From the First Hour of Life: Making the Case for Improved Infant and Young Child Feeding Everywhere.

[2] Baker J.L., Gamborg M., Heitmann B.L., Lissner L., Sørensen T.I.A., Rasmussen K.M. (2008). Breastfeeding reduces postpartum weight retention. Am. J. Clin. Nutr..

[3] Binns C., Lee M., Low W.Y. (2016). The Long-Term Public Health Benefits of Breastfeeding. Asia Pac. J. Public Health.

[4] World Health Organization (2003). Global Strategy for Infant and Young Child Feeding.

[5] Lackey K.A., Fehrenkamp B.D., Pace R.M., Williams J.E., Meehan C.L., McGuire M.A., McGuire M.K. (2021). Breastfeeding Beyond 12 Months: Is There Evidence for Health Impacts?. Annu. Rev. Nutr..

[6] Victora C.G., Bahl R., Barros A.J.D., França G.V.A., Horton S., Krasevec J., Murch S., Sankar M.J., Walker N., Rollins N.C. (2016). Breastfeeding in the 21st century: Epidemiology, mechanisms, and lifelong effect. Lancet.

[7] Farwell A.L. (2017). Integrative Review of Breastfeeding Duration and Influencing Factors Among Women Serving Active Duty in the U. S. Military. J. Obstet. Gynecol. Neonatal Nurs..

[8] Joshi A., Trout K.E., Aguirre T., Wilhelm S. (2014). Exploration of factors influencing initiation and continuation of breastfeeding among Hispanic women living in rural settings: A multi-methods study. Rural Remote Health.

[9] Mendes M.S., Schorn M., Santo L., Oliveira L.D., Giugliani E.R.J. (2021). Factors associated with breastfeeding continuation for 12 months or more among working mothers in a general hospital. Cienc. Saude Coletiva.

[10] Muelbert M., Giugliani E.R.J. (2018). Factors associated with the maintenance of breastfeeding for 6, 12, and 24 months in adolescent mothers. BMC Public Health.

[11] Sağlam N., Bülbül L., Kazancı S.Y., Hatipoğlu S.S. (2019). Factors Affecting Breastfeeding and Complementary Feeding Choices for Children Aged 24 to 48 Months. Sisli Etfal Hastan. Tip Bul..

[12] Sencan I., Tekin O., Tatli M.M. (2013). Factors influencing breastfeeding duration: A survey in a Turkish population. Eur. J. Pediatr..

[13] Wallenborn J.T., Perera R.A., Wheeler D.C., Lu J., Masho S.W. (2019). Workplace support and breastfeeding duration: The mediating effect of breastfeeding intention and self-efficacy. Birth.

[14] Li L., Ye R., Sun C., Wang Q., Wu Y., Lei P., Meng S., Du Y., Li L., Zhou H. (2021). Status and influencing factors of breastfeeding behavior in rural areas of Han, Tibetan and Yi nationalities in Sichuan Province. Wei Sheng Yan Jiu = J. Hyg. Res..

[15] Li S., Yue A., Abbey C., Medina A., Shi Y. (2019). Breastfeeding and the Risk of Illness among Young Children in Rural China. Int. J. Environ. Res. Public Health.

[16] Yang Z., Lai J., Yu D., Duan Y., Pang X., Jiang S., Bi Y., Wang J., Zhao L., Yin S. (2016). Breastfeeding rates in China: A cross-sectional survey and estimate of benefits of improvement. Lancet.

[17] Feng Y., Zhou H., Wang X., Zhang J., Wang Y. (2012). Study of the infant and young child feeding practice in some areas of China and across-country comparison. Chin. J. Child Health Care.

[18] (2018). Protecting, Promoting and Supporting Breastfeeding in Facilities Providing Maternity and Newborn Services: Implementing the Revised Baby-Friendly Hospital Initiative 2018 Inplementation Guidance.

[19] Li C. (2012). Study on the State of Infant Family Rearing. Ph.D. Thesis.

[20] Guo J., Wu H., Fan R., Cai Y., Luo X., Wang X. (2009). Situation of Infant Breastfeeding. J. Prev. Med. Inf..

[21] Wang J., Li N., Xie S., Yang S., Zheng X., Zhang J. (2013). Duration of breastfeeding and its relevant influencing factors on under 2-years-old in rural areas of 10 provinces in China. Chin. J. Epidemiol..

[22] Li P., Liu H. (2017). Breastfeeding duration of children in China—Based on the methods for survival date analysis. Popul. Dev..

[23] Liu S., Li J., Gong C., Cheng M., Song Y. (2014). Evaluation of the feeding status of infants and young children under 2 years old in rural areas of Hubei province. Chin. J. Prev. Med..

[24] Liu J., Xu Y. (2018). Discussion on the best child-bearing age. Chin. J. Woman Child Health Res..

[25] Yuan Z., Min G., Yu H., Zhou X. (1996). Comprehensive evaluation of the optimal childbearing age of primipara. Chin. J. Obstet. Gynecol..

[26] Hu S., Wang X., Li Z., Tao X. (2020). Systematic review of factors reltated to the duration of breastfeeding in China. Chin. J. Child Health Care.

[27] Wang N., Chen Q. (2017). Study on Factors of Duration of Infants’ Breastfeeding: Based on Questionnaire Survey of Yuexiu District of Guangzhou City. Chin. J. Soc. Med..

[28] Su S., Qu W., Ni B., Sun J. (2017). Status quo and influencing factors of breastfeeding among infants aged 6-month and elder in Dalian municipality. Chin. J. Public Health.

[29] Su J., He Z., Wei F., Qin C., Xu Z., Qin X., Lu H. (2019). Duration of breastfeeding and its relevant influencing factors in Guigang. Appl. Prev. Med..

[30] Duan Y., Pan L., Wang J., Yang Z., Xu L., Li J., Wan Q., Liu S., Wan R., Yin S. (2016). Status of exclusive breastfeeding and influencing factors for 1 882 pairs of mother and neonate during 0-7 days postpartum in China. Chin. J. Prev. Med..

[31] Di Manno L., Macdonald J.A., Knight T. (2015). The intergenerational continuity of breastfeeding intention, initiation, and duration: A systematic review. Birth.

[32] Liu W., Li S. (2016). Duration of exclusive breastfeeding and its influence factors in Shandong. Chin. J. Child Health Care.

[33] Kang Y., Yan H., Wang Q., Li Q., Xiao S., Bi Y., Xie H. (2007). The introduction of breastfeeding in children under age of three in the counties of western China in 2005. Chin. J. Epidemiol..

[34] Zhang J., Li M., Zhang H., Zhao H. (2020). Breastfeeding duration and its influencing factors in postpartum women returning to work. Mod. Prev. Med..

[35] Zhang X., Wu D., Ding J., Li X., Gao J., Zhang H. (2019). Duration of breastfeeding and its influencing factors among working women. J. Nurs. Sci..

[36] Li Y., Sun G. (2015). SWOT analysis on the relevant interference factors of breastfeeding. Matern. Child Health Care China.

