# Supplementary material for: Factors Affecting the Breastfeeding Duration of Infants and Young Children in China: A Cross-Sectional Study
Source: Nutrients. 2023 Mar 10;15(6):1353. doi: 10.3390/nu15061353 (PMC10051738; doi:10.3390/nu15061353)
Supplement: Supplementary file 1 [file nutrients-15-01353-s001.zip › nutrients-2220711-supplementary.pdf]

Supplementary Materials

Table S1. Eleven judgment questions used to measure mothers' breastfeeding knowledge (1 point for each, 11 points in total) and their single factor analysis results.

No.	Question	N	Breastfeeding Duration (Months, %)					χ^2	*p* Value
			0-6	6-12	12-18	18-24	over 24		
1	Breastfeeding is beneficial to the child's intellectual and emotional development							8.68	0.003
	No	40	20.0	47.5	25.0	5.0	2.5		
	Yes	961	9.5	38.2	32.0	6.8	13.5		
2	Breast milk will no longer be nutritious after 6 months							5.67	0.017
	No	709	9.0	36.1	34.8	7.9	12.1		
	Yes	292	12.0	44.5	24.3	3.8	15.4		
3	Breast milk can meet the physiological needs of infants at different stages which is unmatched by other milk substitutes							0.13	0.718
	No	92	17.4	28.3	34.8	7.6	12.0		
	Yes	909	9.1	39.6	31.5	6.6	13.2		
4	Breast milk is useless after 1 year old							53.05	<0.001
	No	655	8.5	31.9	34.2	8.2	17.1		
	Yes	346	12.4	51.2	27.2	3.8	5.5		
5	Breastfeeding will make the child less susceptible to allergy							11.22	<0.001
	No	190	12.6	44.2	31.6	6.8	4.7		
	Yes	811	9.2	37.2	31.8	6.7	15.0		
6	The later weaning, the harder it is to wean							13.26	<0.001
	No	359	6.7	33.1	37.9	9.7	12.5		
	Yes	642	11.7	41.6	28.3	5.0	13.4		
7	Do not strictly limit the interval between feedings							24.07	<0.001
	No	307	12.1	47.9	27.0	5.5	7.5		
	Yes	694	8.9	34.4	33.9	7.2	15.6		
8	The child should be allowed to suck the breast as soon as possible after delivery							0.06	0.808
	No	173	8.7	39.3	32.9	4.6	14.5		
	Yes	828	10.1	38.4	31.5	7.1	12.8		
9	Weaning too late will affect the child's eating							0.01	0.914
	No	465	9.5	38.3	34.2	6.2	11.8		
	Yes	536	10.3	38.8	29.7	7.1	14.2		
10	Generally, children can take breast milk until 2 years old before weaning							68.88	<0.001
	No	370	12.2	52.2	28.9	1.6	5.1		
	Yes	631	8.6	30.6	33.4	9.7	17.7		
11	Breastfeeding is beneficial to the recovery of mother's figure and weight loss							10.24	0.001
	No	206	12.6	43.7	32.0	3.4	8.3		
	Yes	795	9.2	37.2	31.7	7.5	14.3		

The standard answer of questions No. 1,3,5,7,8,10,11 is Ture, while No. 2,4,6,9 is False.

Table S2. Sensitivity analysis results for multivariable ordinal logistic regression of influencing factors of breastfeeding duration.

Factor	Full Sample, without Work-Related Variables (n=1001) [a]		Samples with Jobs, with All Variables (n=652) [b]	
	p Value	OR (95%CI)	*p* Value	OR (95%CI)
**Individual Level of Mothers**				
Mother's age (vs ≤25)				
26-30	0.936	1.01(0.78~1.30)	0.002	0.58(0.42~0.81)
≥31	<0.001	0.44(0.28~0.68)	<0.001	0.33(0.19~0.57)
Mother's educational background (vs bachelor or postgraduate)				
Junior college	0.069	0.78(0.60~1.02)	0.527	0.90(0.65~1.25)
Senior high school	0.265	0.79(0.51~1.20)	0.245	0.68(0.35~1.31)
Junior high school or below	<0.001	0.34(0.18~0.62)	0.001	0.21(0.08~0.53)
Mother is a freelancer or full-time mother (vs staff of public institutions and enterprises)	<0.001	1.79(1.36~2.34)	-	-
Urban (vs rural)	-	-	0.074	1.98(0.94~4.20)

Mother's breastfeeding knowledge score (out of 11)	<0.001	1.14(1.07~1.22)	0.054	1.09(1.00~1.19)
Self-rating of physical health during lactation (out of 10, vs 9-10)				
7-8	-	-	0.342	0.85(0.60~1.19)
≤6	-	-	0.010	0.48(0.27~0.84)
Mother supports breastfeeding for over 24 months (vs not support)	0.002	2.00(1.29~3.10)	0.003	2.34(1.35~4.06)
**Individual Level of Children**				
Child's birth weight (vs normal birth weight)				
Low birth weight (<2500g)	<0.001	2.40(1.54~3.74)	0.062	1.84(0.97~3.49)
Macrosomia (>4000g)	0.254	0.76(0.48~1.22)	0.162	0.66(0.37~1.18)
Second child or above (vs first child)	-	-	0.105	1.46(0.92~2.29)
Cesarean delivery (vs vaginal delivery)	0.068	0.79(0.61~1.02)	0.025	0.69(0.50~0.96)
Time between childbirth and the first infant and mom's skin contact (vs <2 hours)				
2-24 hours	0.435	1.12(0.85~1.47)	-	-
>24 hours	0.015	0.36(0.16~0.82)	-	-
Time between childbirth and the first nipple sucking (vs <2 hours)				
2-24 hours	<0.001	0.58(0.44~0.78)	-	-
>24 hours	0.146	1.45(0.88~2.39)	-	-
Child's age when the milk bottle was first used (vs <4 months)				
4-6 months	<0.001	2.24(1.70~2.95)	<0.001	2.15(1.53~3.01)
>6 months	<0.001	2.16(1.53~3.06)	<0.001	2.20(1.40~3.45)
Child's age when supplementary food was added (vs <4 months)				
4-6 months	0.100	1.39(0.94~2.06)	-	-
>6 months	0.034	1.59(1.04~2.43)	-	-
**Family Level**				
Monthly household income per capita (vs <702 USD)				
702-1405 USD	0.266	1.18(0.88~1.59)	-	-
>1405 USD	<0.001	1.99(1.42~2.80)	-	-
Whether the child's father supports breastfeeding for over 24 months (vs not support)				
Uncertain	0.045	1.82(1.01~3.26)	0.332	1.56(0.63~3.87)
Support	0.010	1.79(1.15~2.79)	0.034	1.89(1.05~3.40)
Whether the elderly in the family supports breastfeeding for over 24 months (vs not support)				
Uncertain	-	-	0.532	1.29(0.59~2.82)
Support	-	-	0.051	0.65(0.42~1.00)
**Social Support Level**				
Once received professional breastfeeding education during pregnancy	0.072	0.77(0.58~1.02)	0.004	0.57(0.38~0.84)
Whether maternity leave paid (vs full pay)				
Partially paid	/	/	0.486	0.89(0.65~1.23)
No pay	/	/	0.015	2.71(1.21~6.06)
Continuous breastfeeding after returning back to work	/	/	<0.001	5.09(3.09~8.40)
Had one hour of breastfeeding time every working day	/	/	0.057	1.71(0.99~2.98)
A nursing room was set up in the workplace or nearby area	/	/	0.035	0.68(0.47~0.97)
Had an experience of giving up breastfeeding due to the inconvenience of breastfeeding in public	0.007	1.50(1.12~2.02)	<0.001	2.30(1.54~3.45)
Agreed with the statement that "infant formula has higher nutritional value than breast milk"	-	-	0.006	0.60(0.42~0.86)

OR: adjusted odds ratio; CI: confidence interval; USD: United States dollar. [a] McFadden's R^2=0.11. [b] McFadden's R^2=0.14. - for variables without statistical significance that were removed by stepwise regression; / for not applicable.
